# Editorial: Contemporary Strategies in the Management of Civilian Vascular Trauma

**DOI:** 10.3389/fsurg.2018.00043

**Published:** 2018-06-11

**Authors:** Efthymios D. Avgerinos, Emmanouil Pikoulis

**Affiliations:** ^1^Division of Vascular Surgery, University of Pittsburgh Medical Center, Pittsburgh, PA, United States; ^2^1st Department of Surgery, University of Athens School of Medicine, National and Kapodistrian University of Athens Medical School, Athens, Greece

**Keywords:** civialian trauma, REBOA, vascular trauma, arterial injury, venous injury, aortic injury

Trauma remains the leading cause of death in the 15- to 44-year-old age group in the Western World, as a consequence of a motor vehicle accident, unintentional injury, terrorism, homicide, and suicide. One-third of the patients are dying from exsanguination driven by major vascular trauma and a lot more suffer a limb loss (1, 2). Military experience and new technologies, have altered the overall management of both peripheral and truncal vascular trauma. This research topic presents, through seven state-of the-art reviews, the contemporary management of civilian vascular trauma.

Bacoyiannis et al summarizes the systematic management of abdominal vascular trauma based on anatomical location and type of injury (Karaolanis et al.). Karaolanis et al discusses neck vascular injuries and Patelis et al focuses on special concerns for aortic isthmic injuries (Karaolanis et al.; Patelis et al.). Abou Ali et al. covers the role of vascular shunts in contemporary trauma care, Giannakopoulos et al the treatment alternatives for venous injuries and Ptohis et al the role of embolization (Abou Ali et al.; Giannakopoulos and Avgerinos; Ptohis et al.). Finally, Pikoulis et al. binds all reviews together, starting from the scene ending at the operating room, in the context of damage control at all levels (Pikoulis et al.).

As emerging from this research topic, given the unpredictable nature and wide range of vascular injuries we encounter, having a trauma team, including a vascular surgeon and a diverse array of devices, is key to successful management.

While open surgical management has always been the gold standard, the use of endovascular techniques is becoming more prominent for selected indications. This trend is in part due to advancements in numerous technologies such as embolization materials and covered stents from the aorta to the periphery (Abou Ali et al.; Giannakopoulos and Avgerinos; Karaolanis et al.; Karaolanis et al.; Patelis et al.; Ptohis et al.). Alongside, damage control, novel hemostatic agents, transfusion protocols as well as evolution in the intensive care field have shifted management towards more sophisticated strategies. Finally, hybrid operating rooms offer several advantages to using portable C-arms, particularly for complex endovascular aortic or cerebrovascular procedures and can be integrated into the trauma pathway as an alternative to a traditional operating room. The hybrid room can be useful with concomitant orthopedic injuries as well as visceral bleeding amenable to embolization

Overall, early recognition and control of bleeding can achieve better outcomes. Early management of vascular injury, starting in the field, is imperative for survival no less than any operative maneuver. Fluid resuscitation within the limits of permissive hypotension and appropriate use of tourniquets are part of a damage control response concept that bond with damage control surgery to guarantee optimal outcomes (Pikoulis et al.).

The decision for endovascular or open intervention can be difficult and should be guided by the patient’s clinical condition and anatomy of the injury. When patients are unstable, there is little debate that open surgery takes precedence; however, in the reasonably stable patient, the traditional thinking of mandatory surgical exploration is now challenged (3). Blunt traumatic vascular injuries are largely amenable to endovascular therapy, and some previously high-mortality, penetrating injuries like those to the visceral vessels can now sometimes be successfully treated with covered stenting. Medical management and observation have been shown to be effective for a range of arterial and venous injuries, particularly subcentimeter intimal flaps or intramural hematomas. Thoracic aortic as well as axillosubclavian injuries are associated with favorable endovascular outcomes and have the most robust data supporting their use (Giannakopoulos and Avgerinos; 3). Ideally a multidisciplinary team is required to make appropriate treatment decisions and weigh the risks and benefits of available treatment modalities.

The advent of resuscitative endovascular balloon occlusion of the aorta (REBOA) has increased the interest of the emergency medicine, critical care, and trauma surgery communities in learning endovascular skills. Use of REBOA has been shown to improve hemodynamics, increase survival as compared to historical controls who underwent thoracotomy, and preserve neurological outcome in survivors. Access site complications and limb-related adverse events remain low, and the use of smaller systems (7 Fr) may aid in increasing safety and consideration for use in ambulances or helicopters (Pikoulis et al.).

As the future unfolds, the benefits and detriments of these contemporary strategies are constantly reevaluated targeting optimal care, minimal limb and life losses.

## Author Contributions

EA and EP contributed equally to this manuscript.

## Conflict of Interest Statement

The authors declare that the research was conducted in the absence of any commercial or financial relationships that could be construed as a potential conflict of interest.
